# Ductal activation of oncogenic KRAS alone induces sarcomatoid phenotype

**DOI:** 10.1038/srep13347

**Published:** 2015-08-20

**Authors:** Yong Fu, Zobeida Cruz-Monserrate, H. Helen Lin, Yiyin Chung, Baoan Ji, Szu-min Lin, Steven Vonderfecht, Craig D. Logsdon, Chien-Feng Li, David K. Ann

**Affiliations:** 1 Department of Diabetes and Metabolic Diseases Research, Beckman Research Institute, City of Hope, Duarte, CA 91010, USA; 2 Department of Cancer Biology, UT MD Anderson Cancer Center, Houston, Texas 77054, USA; 3 Molecular and Cellular Biology, Beckman Research Institute, City of Hope, Duarte, CA 91010, USA; 4 Mayo Clinic, Rochester, Minnesota; 5 Shared Research Animal Resources, Beckman Research Institute, City of Hope, Duarte, CA 91010, USA; 6 Department of Pathology, Chi-Mei Medical Center, Tainan, Taiwan; 7 Irell & Manella Graduate School of Biological Sciences, Beckman Research Institute, City of Hope, Duarte, CA 91010, USA

## Abstract

Salivary duct carcinoma (SDC) is an uncommon, but aggressive malignant tumor with a high mortality rate. Herein, we reported the detection of somatic *KRAS* A146T and Q61H mutations in 2 out of 4 (50%) sarcomatoid SDC variants. Transgenic mice carrying the human oncogenic KRAS^G12V^, which spatiotemporal activation by tamoxifen (TAM)-inducible Cre recombinase *Ela-CreERT* in the submandibular gland (SMG) ductal cells, was established and characterized. Visible carcinoma was detected as early as day-15 following oncogenic KRAS^G12V^ induction alone, and these tumors proliferate rapidly with a median survival of 28-days accompanied with histological reminiscences to human sarcomatoid SDC variants. Moreover, these tumors were resistant to cetuximab treatment despite augmented EGFR signaling, attesting its malignancy. Our findings suggest that *LGL-KRas*^*G12V*^*;Ela-CreERT* transgenic mice could serve as a useful preclinical model for investigating underlying mechanisms and developing potential therapies.

Human salivary duct carcinoma (SDC) is a highly aggressive adenocarcinoma of salivary glands. SDC patients usually already have advanced tumor with cervical lymph node involvement when they are diagnosed. Distant metastasis is frequent and is the major cause of death. A key histological feature of SDC is its resemblance to the ductal carcinoma of breast^1^,^2^. Another feature of SDC is its apocrine morphology^3^,^4^. Although SDCs account for less than 3% of all salivary gland tumors, it is an extremely aggressive malignant tumor with a high mortality rate^5^,^6^. Conventional treatments, such as surgery with or without radiotherapy, usually lead to high recurrence rate, and the overall survival is poor with most SDC patients dying within 3-years after diagnosis^7^. Despite the need for improved treatment methods, molecular characterization of SDC falls behind compared to the common malignancies because of its rarity. Recently, genetic aberrations in SDC were reported, including *PIK3CA* mutation^3^,^8^,^9^, mutations in *KRAS, HRAS, and BRAF*^3^,^10^,^11^, amplifications of *ERBB2*^3^,^10^, *EGFR*^10^, and *KRAS*^10^, translocation/amplification of *PLAG1* and *HMGA2*^12^, and androgen receptor expression^4^,^13^,^14^. Of note, multiple genetic alterations were found in many SDC cases^10^. Meanwhile, morphological and molecular variations in SDC have been reported^4^,^15^,^16^.

In this study, we sought to screen for genetic mutations contribute to the pathogenesis of human SDC. RAS proteins have emerged as key oncogenic regulators in many solid tumors. For example, oncogenic activation of the RAS proteins, through missense mutations, is frequently associated with cell proliferation and survival in a variety of cancers, likely due to the impact of constitutively activated RAS on multiple signaling pathways^17^. According to the COSMICv72 catalog of somatic cancer mutations (http://cancer.sanger.ac.uk/), mutations of all RAS isoforms are ranked among the top 20 mutated genes in human salivary tumors, albeit the incidence is lower than in pancreatic, colorectal and lung cancers^18^. The *KRAS* and *HRAS* point mutation frequencies are 2.5% (10/402) and 6.2% (25/401), respectively, in salivary gland tumors. To characterize the status of *RAS* in SDC, we have observed that 2 out of 4 human sarcomatoid SDC cases (50%) harbored oncogenic *KRAS* mutations. However, whether the acquisition of oncogenic *KRAS* mutation would lead to sarcomatoid SDC remains to be experimentally established.

There is currently no animal model to evaluate SDC progression and test candidate therapies. Other attempts to model salivary gland cancers in the mouse have used the MMTV long terminal repeat promoter and have been limited by a lack of ductal cell specificity^19^,^20^. Therefore, it has been challenging to define the initiation of tumorigenesis. The *cytokeratin 5* promoter/enhancer has also been used to drive mutated KRAS expression, resulting in salivary squamous cell carcinoma^21^. However, the cellular origin, especially acinar or ductal origin, could not be determined because of the ubiquity of cytokeratin 5 in epithelial cells. The *Justy* mutant mouse model produced carcinomas in salivary glands with a latency of 6 months and an incidence of ~25%^22^. The long latency period and low incidence rate have also limited the use of this model. *Second*, many transgenic mouse models require a *p53*- or *PTEN*-deficient background for timely tumorigenesis^23^,^24^. Although the use of combinations of lesions might reveal synergistic effects or dependencies of two genetic lesions, the use of multiple mutations substantially complicates the interpretation.

To address this unmet need for a SDC animal model and to examine the role of oncogenic *KRAS* mutation in SDC pathogenesis, we have established a novel mouse model (*LGL-KRas*^*G12V*^*;Ela-CreERT)*. The mouse model uses the SMG ductal cell-specific *Elastase I* (*Ela*) promoter to drive expression of a tamoxifen (TAM)-inducible form of Cre recombinase (CreERT). Upon TAM gavage, Cre-recombinase excised a *LoxP-GFP/Stop-LoxP* (LGL) element to drive *KRAS*^*G12V*^ expression, thus allowing spatial and temporal activation of oncogenic KRAS^G12V^ in the SMG ductal cells. SMG tumors that resemble human SDCs in both morphology and clinical features were detected within two weeks following activation of oncogenic KRAS^G12V^. Moreover, we found that tumor cells originated from a subpopulation of cells at the abluminal region of the granulated convoluted tubules (GCTs) and striated ducts. To our knowledge, this animal model is the first one with a defined ductal origin of salivary gland malignancy. In addition, the *Ela-CreERT* mouse model is potentially useful for studying the role of other oncogenes or tumor suppressor genes in SDCs by crossing with respective floxed strains. Importantly, our *LGL-KRas*^*G12V*^*;Ela-CreERT* mouse model is a step forward due to its ductal cell specificity, high tumor incidence, and rapid onset.

## Results

### *KRAS* but not *EGFR* mutation was identified in human SDC and associated with sarcomatoid variant

**Figure 1:**
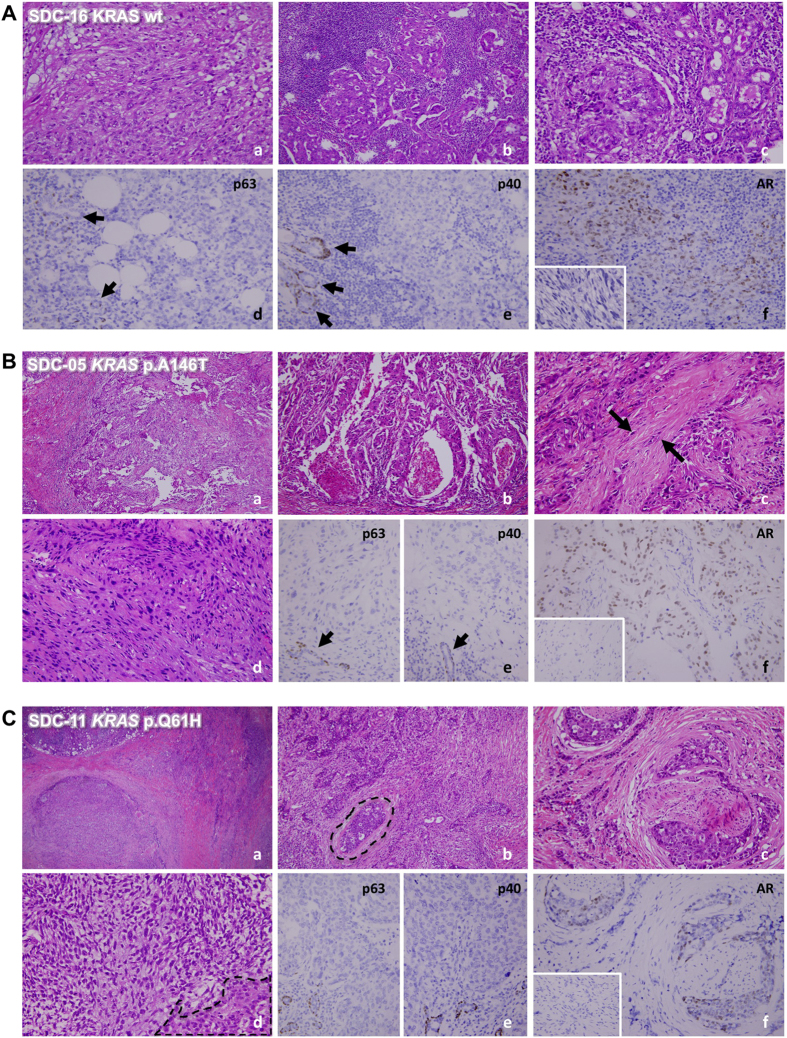
Representative cases of human SDCs. (**A**) A SDC with co-existence of frank sarcomatous change (*a*, 200X) and typical SDC morphology (*b*, 200X). In some of the tumor-adjacent salivary ductules, ductal adenomatoid hyperplasia (*c*, 200X) can be appreciated with focal transformation to carcinoma *in situ*-like lesion, suggesting a hyperplasia-dysplasia-carcinoma pathway and further epithelial-mesenchymal transformation in the pathogenesis of SDC. Immunohistochemically, tumor cells are negative for p63 (*d*, 400X) and p40 (*e*, 400X). The residual salivary ducts served as internal positive control for p63 or p40 (arrows). Although the tumor cells in conventional SDC area were AR-positive (*f*, 400X), the sarcomatoid component was AR-negative (*inset*). (**B**) A SDC with *KRAS* p.A146T mutation shows infiltrative growth (*a*, 100X) by neoplastic glands (*b*, 200X). Foci of desmoplastic reaction entrapping discohesive spindling tumor cells (*c*, 200X, arrows) and frank sarcomatous change (*d*, 400X) are evident. Tumor cells were p63- and p40-negative (*e*, 400X; arrow depicts p63 or p40 positive control). Although the conventional SDC tumor cells were AR-positive (*f*, 400X), tumor cells in the sarcomatous area lost AR expression (*inset*). (**C**) Another SDC featured by *KRAS* p.Q61H mutation also demonstrates infiltrating growth (*a*, 100X) and revealing very focally carcinoma *in situ*-like area embedded in infiltrating tumor nests (*b*, dotted line, 200X). Perineurial invasion (*c*, 200X) and sarcomatous transformation (*d*, dotted line indicates conventional SDC, 400X) are evident. SDC tumor cells were also negative for p63 and p40 (*e*, 400X) and positive to AR (*f*, 400X). Interestingly, as seen in (*B*), sarcomatous areas lost AR expression.

To identify the genetic mutation that contributed to the pathogenesis of SDC, one of the most aggressive subtypes of salivary gland cancers, we investigated 18 SDC biospecimens to search for either somatic *KRas* or *EGFR* mutation. The characteristic morphologic and clinicopathologic features of 18 SDC tumors are shown in Fig. 1 and summarized in Table 1. The mean age at diagnosis of 18 patients, 12 males and 6 females, was 59.1-years (range from 41- to 81-years). All these 18 cases were located in major salivary glands with a preference for parotid gland. All cases received curative surgery and metastatic deposition was identified involving regional lymph nodes (n = 9) or beyond (n = 4). Histologically, 4 cases were classified as sarcomatoid variant, including one that showed both sarcomatoid and micropapillary features, one that demonstrated micropapillary and one with mucinous feature, respectively. To further confirm the diagnosis and exclude other morphological mimickers of SDCs, a large panel of immunohistochemical (IHC) studies, including p63, p40, androgen receptor (AR) and other markers were performed (Fig. 1). Using the OncoFOCUS panel, we detected 2 out of 4 cases of sarcomatoid variant (SDC-5 and SDC-11, Fig. 1B,C) harboring oncogenic KRAS p.A146T (AGC>AAC) and p.Q61H (CAA>CAC) mutations, respectively (Table 1), which were further confirmed by pyrosequencing (Supplementary Fig. S1). All cases were negative for *NRAS*, *EGFR* and *BRAF* mutations. Both cases showing KRAS mutations, A146T and Q61H respectively, exhibited characteristics of sarcomatoid variants (Fig. 1B,C), suggesting an association between KRAS mutation and sarcomatoid change (*p* = 0.005). We noticed that sarcomatoid areas of SDCs were negative for AR expression (Fig. 1), consistent with the report by Nagao *et al*. showing that while the conventional SDC carcinomatous areas were AR-positive, sarcomatoid component exhibited AR-negative phenotype^25^.

### Oncogenic KRAS^G12V^ activation causes SMG tumors

**Figure 2:**
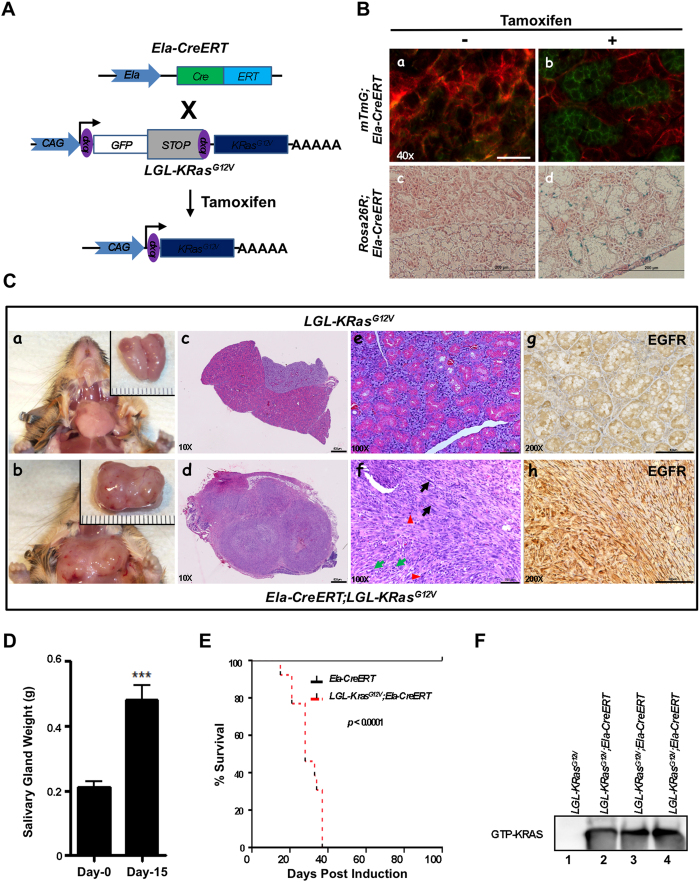
*Elastase I*-driven oncogenic KRAS^G12V^ expression induces SDC-like tumors in the SMGs. (**A**) The diagram of the *Ela-CreERT* and *LGL-KRas*^*G12V*^ transgenes^26^,^30^. Expression of the TAM-inducible Cre recombinase (CreERT) is driven by *elastase I* promoter/enhancer. KRAS^G12V^ expression is blocked by a loxP-GFP-Stop-loxP cassette (LGL). A mouse BAC of 222-kb carrying the intact *elastase I* gene was used to provide the endogenous native context of *elastase I* gene^26^. CreERT removes STOP cassette (LGL) through recombination, allowing the KRAS^G12V^ expression. (**B**) *Elastase I*-driven recombination in mTmG and Rosa26R reporter mice ascertains ductal original. Green fluorescence was exclusively detected in the ductal cells of *mTmG;Ela-CreERT* reporter mice after vehicle (*panel a*) or TAM-feeding (*panel b*). Scale bar: 50 μm (*panel a*). β-galactosidase activity was observed in the SMG ductal cells of *Rosa26R;Ela-CreERT* mice after TAM-feeding (*panel d*) compared to control mice (*panel c*). Scale bar: 200 μm. (**C**) Characterization of SMGs of *LGL-KRas*^*G12V*^*;Ela-CreERT* mice on day-24 post TAM-feeding. Gross anatomy revealed large ventrolateral cervical masses in SMGs of *LGL-KRas*^*G12V*^*;Ela-CreERT* mice on day-24 (*panel b*). H&E and immunohistochemical (IHC) staining showed the microscopic abnormalities in the SMGs of TAM-fed *LGL-KRas*^*G12V*^*;Ela-CreERT* mice on day-24 (*panels d, f, h)*. Scale bars: 800 μm (in *panels c* and *d*); 100 μm (*panels e–h*). (**D**) Wet weight of SMG from *LGL-KRas*^*G12V*^*;Ela-CreERT* mice at 15-days post TAM-feeding (n = 13) or not (n = 7). ***p < 0.001. (**E**) Overall survival of mice after KRAS^G12V^ induction is drastically reduced. Percent survival of *Ela-CreERT* versus *LGL-KRas*^*G12V*^*;Ela-CreERT* mice after TAM administration. Median survival of LGL-KRasG12V;Ela-CreERT mice was 28-days. n = 13 per group; *p* < 0.0001 by Log-rank (Mantel-Cox) test. (**F**) KRAS^G12V^ is activated in the SMG tumors of *LGL-KRas*^*G12V*^*;Ela-CreERT* mice following TAM-gavage. Transgenic mice of indicated genotypes were gavaged with TAM. SMG samples were harvested on day-24 and whole tissue lysates (1 mg) from each sample were pulled down with Raf-1 RBD agarose beads. Pulldown reactions were resolved by SDS-PAGE (12%) and Western blotting was performed with an anti-KRAS antibody to detect active (GTP-bound) KRAS^G12V^.

Next, we sought to establish a mouse model to target oncogenic KRAS expression in salivary glands in order to investigate the mechanisms underlying the role of KRAS mutations in SDC. A TAM-inducible Cre recombinase (CreERT), engineered into the native *elastase I* gene context (*Ela-CreERT*; Fig. 2A) within a mouse BAC (222-kb)^26^, has been demonstrated to induce salivary gland tumor upon crossing with *KRAS*^*G12V*^ mice^27^. We thereby utilized this transgenic mouse model to investigate the underlying mechanisms. To explore the cell type in which Cre-mediated recombination in the SMGs of TAM-fed *LGL-KRas*^*G12V*^*;Ela-CreERT* mice, two independent reporter assays were performed. First, we crossed *Ela-CreERT* mice with *mTmG* reporter mice^28^. Double transgenic *mTmG;Ela-CreERT* mice were fed with vehicle or TAM (3 mg/40g body weight) for 5-days (Supplementary Fig. S2). The fluorescence (GFP)-positive cells, a result of removing *mT* and activating *mG* by Cre recombinase, were barely seen in the SMGs of vehicle-fed mice (Fig. 2B, *panel a*). In contrast, GFP-bearing cells were exclusively detected in ducts, mainly in GCTs, with a background of red fluorescence-positive acinar cells in the SMGs of TAM-fed *mTmG;Ela-CreERT* mice (Fig. 2B, *panel b*). Likewise, *Ela-CreERT* mice were crossed with the *Rosa26R* reporter mice^29^. In these double-transgenic *Rosa26R;Ela-CreERT* mice, β-galactosidase activity was induced in the SMG ductal cells upon TAM-feeding (Fig. 2B, *panel d*). These observations confirmed that TAM gavage turned on Cre-recombination in ductal cells of mice with *Ela-CreERT* allele.

On day-15 post TAM-administration, *LGL-KRas*^*G12V*^*;Ela-CreERT*, but not *LGL-KRas*^*G12V*^, mice developed palpable ventrolateral cervical masses, which were felt hard and irregular. Necropsy revealed firm and solid masses in the SMGs. Nearly 100% of TAM-fed *LGL-KRas*^*G12V*^*;Ela-CreERT* mice exhibited large ventrolateral cervical masses, ranged between 10- to 15-mm in diameter, irrespective of gender on day-24 (Fig. 2C, *panels a–d*). While cysts were occasionally found in the tumors of some mice, these tumors proliferated at a fast rate. Masses were up to >2× bigger in wet weight than their counterparts from control mice on day-15 (Fig. 2D), attesting their fast tumor expansion. H&E staining showed that the normal glandular and ductal architectures of SMGs were barely noticeable on day-24 (Fig. 2C, *panels d, f*). Instead, the SMGs were occupied by the highly cellular and multi-nodular masses encapsulated by fibrous septa. As shown in Fig. 2C (*panel f*), the masses contained highly atypical hyperchromatic spindle or rhabdoid cells arranged in a fascicular manner. Multinucleate, large bizarre cells with a high nuclear to cytoplasm ratio (Fig. 2C, *panel f*, red arrowheads) were scattered among atypical spindle cells (Fig. 2C, *panel f*, black arrows). Mitotic figures (Fig. 2C, *panel f*, green arrows) were common. Positive staining of CD-45, Gr-1, and F4/80, respectively, clearly supported the infiltration of inflammatory cells (e.g., leucocytes and macrophages) in SMGs on day-15 (Supplementary Fig. S3A). Consistent with p63, p40 and AR-status in human SDCs (Fig. 1B,C), these oncogenic KRAS-expressing sarcomatoid tumor cells were negative for the expressions of these markers (Supplementary Fig. S3B). In addition, IHC studies with an antibody recognizing the epidermal growth factor receptor (EGFR) showed that EGFR signal was up-regulated in the tumors compared with normal SMG tissues (Fig. 2C, *panels g, h*).

All tumor-bearing *KRas*^*G12V*^*;Ela-CreERT* mice, compared to TAM-fed *Ela-CreERT* mice, died within 40-days after tumor induction (Fig. 2E). Necropsy revealed no gross abnormality in other organs; however, pancreatic fibrosis was observed as reported previously^30^. Biochemically, active (GTP-bound) RAS pulldown assays revealed that all tumorous SMG tissues from three randomly selected, TAM-fed *LGL-KRas*^*G12V*^*;Ela-CreERT* mice exhibited activated KRAS (Fig. 2F, *lanes 2–4*). In contrast, no GTP-bound KRAS was detected from apparently normal SMGs of a TAM-fed *LGL-KRas*^*G12V*^ mouse (Fig. 2F, *lane 1*). Altogether, we concluded that the observed tumors originated from oncogenic KRAS activation. Given that these tumors frequently infiltrated adjacent sublingual glands, and the division between SMG lobes was indistinguishable (Fig. 2C, *panel d*), the whole SMG masses were prosected *en bloc* for fixation and subsequent studies. Because the median survival of the tumor-bearing mice was 28-days (Fig. 2E), we set day-24 as the terminal point for most of our studies (Supplementary Fig. S2).

### KRAS^G12V^-driven SMG tumors exhibit SDC-like characteristics

**Figure 3:**
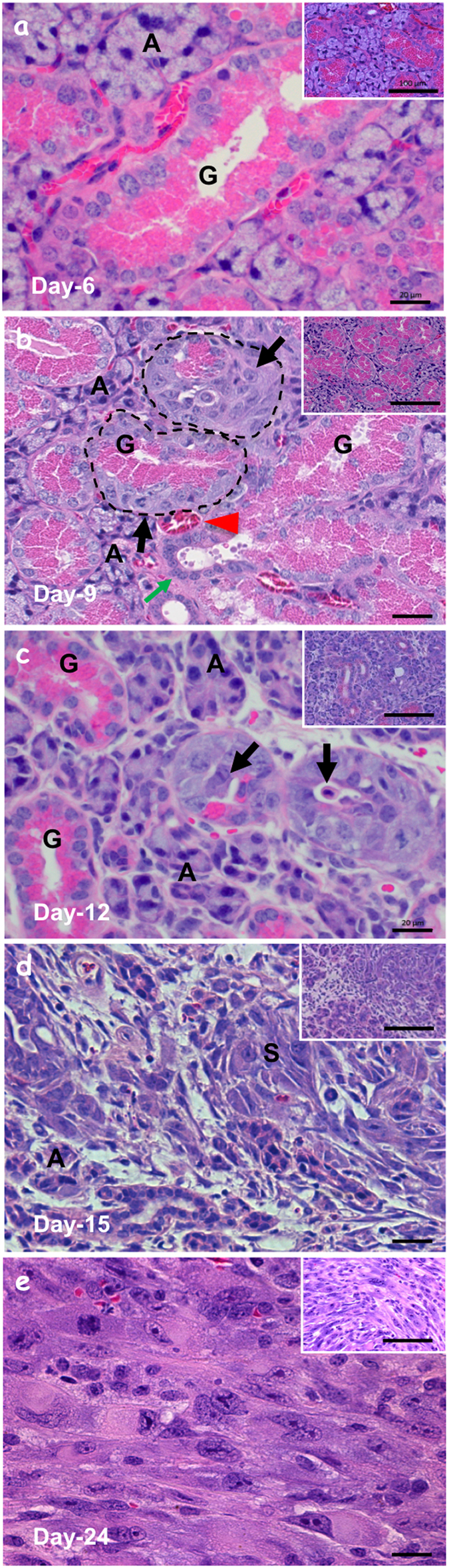
KRAS^G12V^-induced tumors exhibit SDC-like phenotypes. (**A**–**E**) H&E staining of submandibular glands harvested from *LGL-KRas*^*G12V*^*;Ela-CreERT* mice at indicated time points revealed gradual abnormal manifestations starting from day-9 post TAM-feeding. Scale bars: 20 μm and100 μm (*inset*). G: GCT; A: acini; S: sarcomatous element; SD: striated duct; ED: excretory duct.

To characterize the KRAS^G12V^-mediated tumorigenesis, SMG tissues from *LGL-KRas*^*G12V*^*;Ela-CreERT* and *LGL-KRas*^*G12V*^ mice were harvested at various times (day-6, -9, -12, -15 and -24) following induction, respectively (Supplementary Fig. S2). Almost no microscopic abnormality of either GCTs (labeled G) or acini (labeled A) in the SMGs was detected by H&E staining on day-6 (Fig. 3, *panel a*). On day-9, while most acinar and ductal structures appeared normal and the basement membranes were intact (Fig. 3, *panel b, inset*), higher magnification revealed sporadic dysplasia (Fig. 3, *panel b*, black arrows) in GCTs near a striated duct (green arrow) in the proximity to blood vessel (red arrowhead), but the basement membranes were still intact (dotted lines). Higher magnification images revealed atypical giant cells (black arrow) arose between normal GCTs (G) and the bordering intact basement membrane (Supplementary Fig. S4A, *panel a*). The striated duct (SD) of SMGs also developed dysplasia with multiple layers and giant and bizarre nucleus on day-9 (black arrow, Supplementary Fig. S4A, *panel b*). Transition from hyperplasia (green arrow) to dysplasia (black arrow) was noticed in an excretory duct (ED) of SMGs on day-12 (Supplementary Fig. S4A, *panel c*). Hyperplasia and dysplasia were located at the abluminal side of the epithelium. Ductal *in situ* carcinoma was observed on day-12 (yellow dashed line, Supplementary Fig. S4A, *panel d*). *In situ* carcinoma and dysplasia were frequently detected and some lumens were filled with membranous elements on day-12 (black arrows, Fig. 3, *panel c*). Although the expanding ductal cells compressed the adjacent acini and distorted some of their organization, the overall morphology of acinar cells appeared normal (Fig. 3, *panel c*). In some abnormal ducts, the basement membrane was broken and microinvasive carcinoma formed on day-12 (red dashed line, Supplementary Fig. S4A, *panel e*). The invasion (black arrow) was prominent and the residual ductal structure (yellow dashed line) was barely observable on day-12 (Supplementary Fig. S4A, *panel f*).

Most SMGs were occupied by masses of malignant cells on day-15 (Fig. 3, *panel d*). Residual acini (labeled A) were scattered among these masses. Sarcomatous elements (labeled S) were composed of spindle or rhabdoid cells without any discernible structures. In addition, these tumors became fibrotic as shown by the increase in Masson’s trichrome staining of abnormal collagen deposition throughout the SMGs (Supplementary Fig. S4B). Increased extracellular matrix production (Supplementary Fig. S4B) and marked infiltration of inflammatory cells (Supplementary Fig. S3A) underscored an altered SMG microenvironment. On day-24, the whole field was occupied with spindle or rhabdoid cells arranged in fascicular pattern without forming any structures, exhibiting typical sarcomatous change (Fig. 3, *panel e*).

All these observations suggested that high degree dysplasia in ductal epithelium (Supplementary Fig. S4A, *panel d*), microinvasive carcinoma (Supplementary Fig. S4A, *panel e*), and invasive carcinoma (Supplementary Fig. S4A, *panel f*) co-existed with some normal GCTs or residual GCTs with reduced eosin-stained area, mimicking human SDC (Fig. 1). Notably, the area of eosin-stained granular region gradually decreased starting from day-9 (Fig. 3A, *panels b, c, d, e, insets*). Interestingly, dysplasia observed on day-9 were preferentially located at GCTs or striated ducts in the close proximity to a dyad structure—a striated duct accompanied by one or more blood vessels (Fig. 3A, *panel d*, and Supplementary Fig. S5). Lastly, necropsy also revealed abnormalities in parotid and sublingual glands (Supplementary Fig. S6). However, the frequency of abnormal parotid and sublingual glands and their magnitude of tumor expansion, compared to SMGs, were markedly reduced. At this moment, we cannot differentiate whether these were *de novo* tumors or disseminated from SMGs. Nonetheless, we mainly focused on SMG tumors in the remaining studies. Altogether, consistent with previous *in vivo* reporter assays (Fig. 2B), the early morphologically abnormal cells were detected in the ductal components (Fig. 3 and Supplementary Fig. S4,S5).

### KRAS^G12V^-driven SDC-like tumors originate from SMG ductal cells

**Figure 4:**
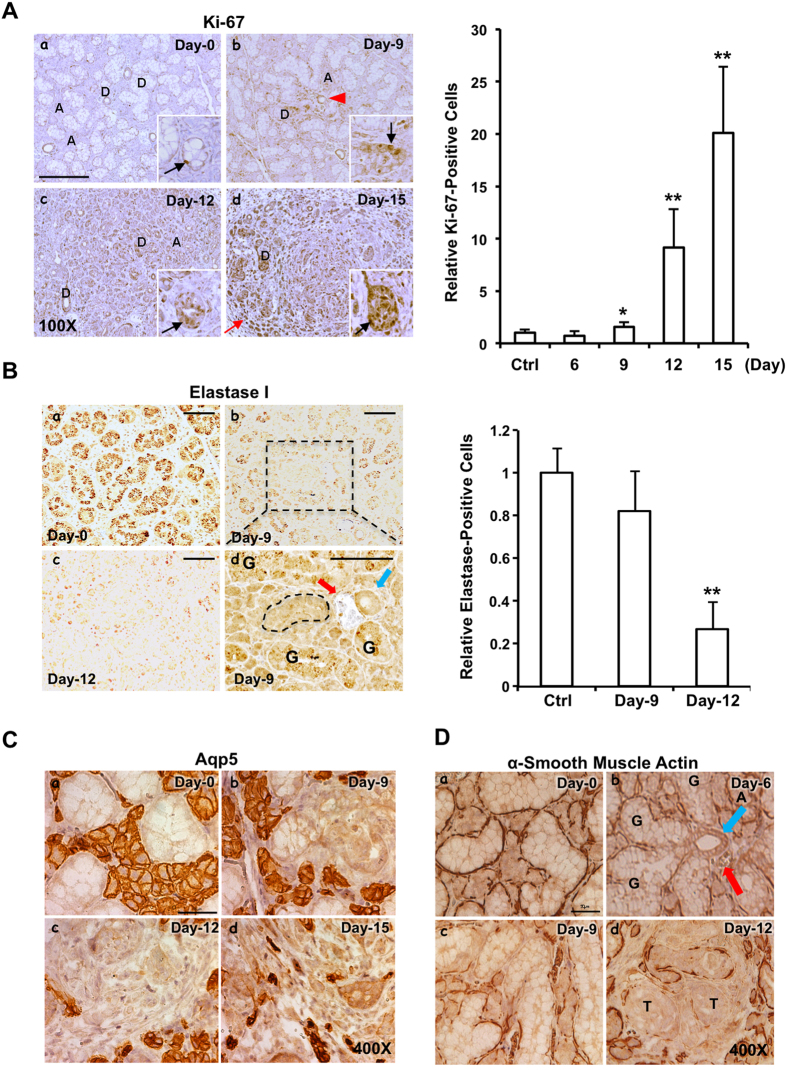
Cell proliferation starting from day-9 following oncogenic KRAS activation. (**A**) Nuclear Ki-67-positive cells increased mainly at the ductal cells starting from day-9 post KRAS^G12V^ induction. Scale bars: 250 μm; *inset*: 200X; A: acini; D: duct; black arrow: nuclear Ki-67-positive ductal cell; red arrow: nuclear Ki-67-positive fibroblast; red arrowhead: blood vessel. The relative fold change in the nuclear Ki-67-positive staining with the level in the SMGs of *LGL-KRas*^*G12V*^*;Ela-CreERT* mice prior to Tam-feeding (Ctrl) set as 1. Results are shown as mean ± S.D. enumerated from 6 randomly selected non-overlapping fields (10X) by Image-Pro Premier 9.0; *p < 0.05, **p < 0.01. (**B**) Elastase I accumulation in the SMG ductal cells of *LGL-KRas*^*G12V*^*;Ela-CreERT* mice decreased during the course of tumorigenesis (*left panels*). *Panel d* (enlarged from boxed region in *panel b*): elastase I staining was lost in a GCT (shown by dotted line, *panel d*) near a dyad structure of a striated duct (blue arrow) and a blood vessel (red arrow); residual elastase I staining was visualized in some GCTs (label with G). Scale bar: 100 μm (*panels a–c*:); 100 μm (*panel d*, enlarged from *b*). The relative fold change in the elastase I-positive staining (*right panel*). Quantitative analyses of elastase I-positive staining with level at control SMGs set as 1. Results are shown as mean ± S.D. enumerated from 5 randomly selected non-overlapping fields (10X) by Image-Pro Premier 9.0; **p < 0.01. (**C**) Loss of AQP5 staining during KRAS^G12V^-induced tumorigenesis. Immunochemical staining with an anti-AQP5 antibody in the SMGs of *LGL-KRas*^*G12V*^*;Ela-CreERT* mice at indicated time points following TAM-feeding. Scale bar: 50 μm. (**D**) Gradual loss of α-smooth muscle actin (αSMA) expression during early tumorigenesis. The earliest loss of αSMA occurred on day-6 in GCTs near a dyad structure of a striated duct (blue arrow) and a blood vessel (red arrow). Scale bar: 50 μm; G: GCT; T: tumor cell masses.

To characterize the rapid expansion nature of KRAS^G12V^-driven SMG tumors, IHC studies with an antibody recognizing Ki-67, a surrogate proliferation index, was performed at sections collected at various times (day-0, -6, -9, -12 and -15) post induction with TAM. In general, less than 3% cells were nuclear Ki-67-positive (black arrow) and sparsely distributed in both ductal and the acinar compartments on day-0 (Fig. 4A, *panel a*). A notable increase in nuclear Ki-67-positive staining started from day-9 and continued through day-15 (Fig. 4A, *right panel*). These positive staining was detected in the ducts, mainly GCTs, while positive staining in acini remained largely undetectable (Fig. 4A, *left panel b*). Further, the increased nuclear Ki-67 staining (day-9) was not evenly distributed through the ductal system; instead, nuclear Ki-67 staining was enriched in regions in the close proximity to the structure consisting of striated duct accompanied by blood vessel (Fig. 4A, *left panel b*, red arrowhead). Consistently, Supplementary Fig. S7 shows that the early nuclear Ki67 staining preferentially occurred in the close proximity to blood vessel (day-9, red arrowhead: blood vessel, green arrow: striated ducts, black arrow: Ki67-positive). Notably, most nuclear Ki-67-positive signals were located at both the abluminal side of the aberrantly proliferating cells associated with GCTs and other types of ducts and the tumor masses on day-12 and day-15 (Fig. 4A, *left panels c, d*), supporting the expansion of these tumorous cells. Nuclear Ki-67-positive staining was also observed in the fibrous tissues between tumor masses (Fig. 4A, *left panel c*, red arrow).

Next, IHC staining of elastase I and aquaporin 5 (AQP5, an acinar marker) was performed to further validate the ductal origin of SMG tumors. As expected, granular elastase I-positive staining was visible at almost all GCTs on day-0 (Fig. 4B, *left panel a*), then gradually weakened at some GCTs on day-9 (Fig. 4B, *left panels b, d*), and eventually became diffused and sparse or undetectable on day-12 (Fig. 4B, *left panel c*). Notably, a GCT (black dotted line, Fig. 4B, *left panel d*) in the close proximity to a dyad consisting of striated duct (blue arrow) and blood vessel (red arrow) had completely lost granular elastase I staining, consistent with the disappearance of eosin-stained secretory granules in H&E staining (Fig. 3 and Supplementary Fig. S4A). Quantification on the gradual loss of elastase I staining during the course of tumorigenesis is shown in Fig. 4B (*right panel*). Altogether, these results suggested that selected elastase I-expressing duct cells were transformed by KRAS^G12V^ and lost their elastase I expression in the SMGs of the TAM-administered *LGL-KRas*^*G12V*^*;Ela-CreERT* mice. In contrast, AQP5-positive cells were restricted to acini of SMGs in the *LGL-KRas*^*G12V*^*;Ela-CreERT* mice prior to and post TAM-administration (Fig. 4C). In parallel, these AQP5-positive acini were compressed and distorted and AQP5-positive staining signals decreased steadily in the acini from day-6 to -15 (Fig. 4C, *panels b–d*). These observations suggested that KRAS^G12V^-transformed elastase I-expressing GCT cells rapidly expanded and outcompeted acinar cells during the course of SMG tumorigenesis of *LGL-KRas*^*G12V*^*;Ela-CreERT* mice.

Lastly, IHC staining with an antibody recognizing α-smooth muscle actin (αSMA) was performed to profile the molecular changes during the course of tumorigenesis. As shown in Fig. 4D, IHC revealed an αSMA-positive signal surrounding both ducts and acini of SMGs of *LGL-KRas*^*G12V*^*;Ela-CreERT* mice prior to TAM-feeding (day-0, *panel a*). In contrast, a very small percentage of GCTs adjacent to a dyad structure of striated duct (blue arrow) and blood vessel (red arrow) lost αSMA staining on day-6 (Fig. 4D, *panel b*), prior to the appearance of nuclear Ki-67 on day-9 (Fig. 4A, *left panel b*). Gradually, more GCTs, located outside the regions in the close proximity to the dyad structure of striated duct and blood vessel, lost peripheral αSMA staining on day-9 and day-12 (Fig. 4D, *panels c, d*). Remaining αSMA staining was restricted to the residual acini and ducts; no αSMA staining was detected in the tumorous masses (Fig. 4D, *panel d*). Based on all results of H&E staining, nuclear staining of Ki-67, elastase I promoter-driven reporter expression, and αSMA staining, we concluded that the tumorous cells were of ductal origin and acinar cells were not transformed at least in the early stage of the KRAS^G12V^-driven SMG tumorigenesis. Moreover, we postulated that the earliest tumorigenesis sign was the loss of αSMA in the GCTs around striated ducts accompanied with blood vessels (day-6), then followed by dysplasia and accompanied with nuclear Ki-67 positive staining.

### Cetuximab-resistance in KRAS^G12V^-driven SMG tumors

**Figure 5:**
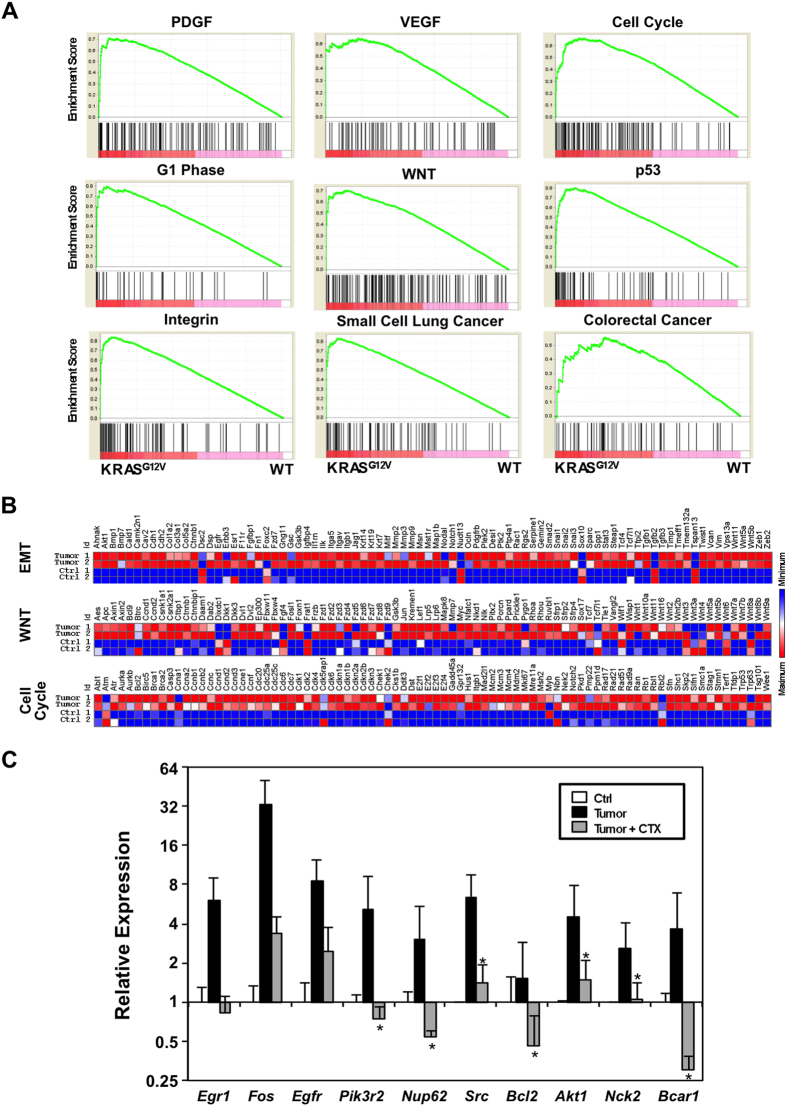
KRAS^G12V^ activates multiple signaling pathways in the tumors. (**A**) Gene Set Enrichment Analysis (GESA) of enriched pathways activated by KRAS^G12V^. Messenger RNA was extracted from tumor samples and normal gland samples for RNA-seq. GESA showed that upregulated genes were enriched in the indicated pathways. *p* < 0.01 and an FDR < 25%. (**B**) PCR array analyses of mRNA showed that genes in EMT, WNT, and Cell Cycle pathways were upregulated in the tumors compared to normal gland tissue, with *p* < 0.001. (**C**) The effect of EGFR inhibitor cetuximab (CTX) on the signaling pathways activated by KRAS^G12V^. Quantitative RT-PCR showed that multiple components of EGFR pathway were activated in tumors, and these activated components were effectively inhibited by cetuximab. *p < 0.05.

RNA-seq analyses were performed to identify the genes and pathways activated by KRAS^G12V^ in the SMGs of TAM-administered *LGL-KRas*^*G12V*^*;Ela-CreERT* mice. Among 19510 annotated transcripts, 2689 transcripts (13.8%) were up-regulated and 254 transcripts (1.3%) were down-regulated in tumors compared to normal SMGs (prior to TAM-administration) (*p* < 0.01). Gene set enrichment analyses demonstrated that 25 gene sets were enriched in tumor tissues (*p* < 0.01 and FDR < 25% (Fig. 5A)). These enriched gene sets include signaling pathways of PDGF, VEGF, integrin, p53, WNT, small cell lung cancer, colorectal cancer, and several cell cycle-related (Fig. 5A). Both non-small cell lung cancer pathway^31^,^32^,^33^ and colorectal cancer signaling pathway^34^,^35^ suggested the involvement of EGF/EGFR pathway in KRAS^G12V^-driven tumorigenesis, which was also supported by the increased EGFR signals in tumors (Fig. 2C, *panel h*). To validate the RNA-seq results, we first performed quantitative PCR array analyses of three pathways: Cell Cycle, WNT, and EMT. As shown in Fig. 5B, members of these three pathways were mostly up-regulated in tumors compared to their counterparts (*p* < 0.001), attesting GSEA analyses. Next, gene expression of factors relating to EGFR signaling in tumor samples and their control counterparts were analyzed by quantitative RT-PCR. The result showed that multiple components of EGFR pathway were up-regulated in tumors (Fig. 5C). Although sarcomatoid SDC-like SMG tumors developed in the SMGs of both genders, the mean wet weight of SMG tumors from age-matched male mice (~715 mg) was almost twice that from female mice (~367 mg) (Supplementary Fig. S8A). It has been reported that EGF-producing GCTs constitute 45% of the volume of SMG in adult male mice, while only 12% in female mice^36^. Moreover, EGFR activation has been shown to drive *KRas*^*G12V*^-dependent pancreatic tumorigenesis in both mouse model of pancreatic cancer driven by mutant KRAS and human biopsies^37^,^38^.

Taken together, it is possible that local EGF-rich niche promoted SMG tumor growth. Lastly, we investigated the involvement of EGFR pathway in the KRAS^G12V^-induced SMG tumorigenesis by administering *LGL-KRas*^*G12V*^*;Ela-CreERT* mice with EGFR inhibitor cetuximab (CTX; 1 mg, *i.p*., three times per week) following KRAS^G12V^ induction. Although quantitative PCR array of selected tumor tissues showed that cetuximab effectively inhibited certain transcriptional signatures activated by KRAS^G12V^ (Fig. 5C), SMG tumor development was unaffected (Supplementary Fig. S8B). *In vitro* clonogenic assays demonstrated that cetuximab failed to reduce viability of primary salivary gland tumor cells (Supplementary Fig. S8C). The insensitivities of the salivary gland tumors or tumor cells to cetuximab treatment were not due to the lack of activity of the cetuximab we used, since cetuximab partially inhibited EGF-induced ERK/AKT phosphorylation in serum-starved tumor cells (Supplementary Fig. S8D). However, cetuximab failed to reduce AKT or ERK phosphorylation in the primary tumor cells cultured continually in growth medium (Supplementary Fig. S8E). Altogether, our results were consistent with studies showing that KRAS mutations not only served as a biomarker of intrinsic resistance to EGFR-targeted agents in patients with colorectal cancer^39^, but also were causally responsible for acquired resistance to cetuximab^40^. Thus, despite down-regulated the abundance of a subpopulation of messages (Fig. 5C), KRAS mutation might render SDC-like SMG tumors refractory to cetuximab, also attesting their malignancy.

## Discussion

We have identified oncogenic KRAS mutation in human sarcomatoid SDCs and further generated and characterized a conditional inducible transgenic mouse model, *LGL-KRas*^*G12V*^*;Ela-CreERT*, to demonstrate the associated pathological manifestations. In this model, KRAS^G12V^ activation in the SMG ductal cells alone was sufficient to induce undifferentiated, high-grade SDC-like tumors in the SMGs of transgenic mice within 12-days. Active RAS pulldown assay further provided biochemical evidence on the activated KRAS in tumors of *LGL-KRas*^*G12V*^*;Ela-CreERT* mice (Fig. 2F), supporting that the observed SMG tumors were originated from oncogenic KRAS activation. In this report, we have meticulously documented the morphological changes from the ductal adenomatoid hyperplasia-like lesion to *in situ* carcinoma, microinvasive carcinoma, invasive carcinoma, to sarcomatoid tumorous cells in the SMGs of *LGL-KRas*^*G12V*^*;Ela-CreERT* model (Fig. 3 and Supplementary Fig. S4), reminiscent of human SDCs^5^,^6^ (Fig. 1). Notably, both the mouse SDC-like tumors and the undifferentiated human SDCs presented rhabdoid or spindle cells without discernible glandular structure. Moreover, both are rapidly growing and with frequent infiltration to adjacent tissues. Altogether, our characterizations suggested that the *LGL-KRas*^*G12V*^*;Ela-CreERT* mouse is a useful preclinical model system for studying human SDC, especially for sarcomatoid variants. In addition, many human malignant salivary gland tumors develop from ductal reserve cells^41^. Conceivably, *Ela-CreERT* mice could be a useful tool for conditional submandibular ductal oncogene activation or tumor suppressor inactivation.

There are two unique features distinguishing this *LGL-KRas*^*G12V*^*;Ela-CreERT* mouse SMG tumor model: the short latency period with poor prognosis and the ductal origin, implicating that SMG ducal component might provide a niche to nurture SDC-like tumor development. This possibility was further supported by observations of the early pathological changes, including the loss of αSMA staining, positive nuclear Ki-67 staining, and the loss of elastase I staining, all occurred in regions in the close proximity of dyad structure of striated ducts and blood vessels. We hypothesize that the adjacent blood vessels or striated ducts provided the growth factors as well as other biologically active molecules stored in the eosin-stained granules of ductal cells^42^ to promote the progression of SMG tumors as a microenvironmental niche. First, these molecules might facilitate KRAS^G12V^-expressing cells bypassing oncogene-induced senescence^43^,^44^, which is a key step to mitigate tumorigenesis. Second, local growth factors might contribute to robust and sustained activation of KRAS^45^, and hence its downstream signaling. Third, these molecules could accelerate phenotypic manifestations of SDC-like tumors. Indeed we found that female developed smaller tumors than male mice did from the comparison in same litter (Supplementary Fig. S8A). Moreover, stimulating GCT secretion with an α-adrenergic agonist cyclocytidine leads to a spike in blood growth factor levels and a depletion of growth factors in the eosin-stained granules^46^,^47^ further supported this notion. Lastly, our observations suggest that there is substantial heterogeneity between ductal cells with regard to their propensity to initiate tumorous masses. We speculate that the heterogeneity in the ductal cells reflects, at least in part, the microenvironmental niche exerted by surrounding cells remains to be further investigated.

Interestingly, human SMG EGFR in ductal cells are mainly detected at the basolateral vesicles^48^, supporting that the SMG EGFR pathway was already activated by locally produced ligands. Moreover, our gene expression profile analyses revealed that a significant portion of oncogenic KRAS-increased messages was components of EGFR pathway (Fig. 5A) and IHC showed EGFR over expression in the SDC-like tumors (Fig. 2C), supporting a potential feed-forward regulation. EGFR is known to be frequently overexpressed in 71% of the different subtypes of salivary gland cancer cases^49^ and is currently being considered as a relevant target for treatment^50^,^51^. EGFR inhibitors have been clinically effective for the non-small cell lung cancer^31^,^32^,^33^ and colorectal cancer^34^,^35^. We speculate that EGF/EGFR pathway could promote epithelial to mesenchymal transition^43^ or endow cancer cells with stem-like phenotype^52^ in these KRAS^G12V^-driven SDC-like tumors. Although EGFR inhibitor failed to retard SMG tumorigenesis in our model (Supplementary Fig. S8B), we cannot completely rule out its involvement in tumor initiation.

Lastly, based on our results, we propose that oncogenic KRAS mutation promotes sarcomatous manifestations. According to catalogue of somatic mutations in cancers (http://cancer.sanger.ac.uk/cancergenome/projects/cosmic/) that mutations of all RASs are ranked among the top 20 mutated genes in the salivary gland tumors, albeit the incidence is relatively lower than pancreatic, colorectal and lung cancers^18^. In this report, we showed that KRAS mutation was detected in 2 out 4 (50%) sarcomatoid variants. However, we cannot rule out concurrent mutations, such as in PIK3CA gene^8^, in these cases. Although results from intensive studies have identified various RAS mutations of different RAS members in a myriad of cancer patients and transgenic models, the correlation between the specificity of RAS mutations and the aggressiveness of the disease has not been fully established so far (reviewed in^53^). Emerging evidence further suggest that many different RAS mutations may have a common effect, while the difference in the end points of RAS mutations is caused by the different environmental contexts where RAS mutation occurs^54^,^55^,^56^,^57^.

In summary, to our knowledge, this is the first demonstration of oncogenic KRAS mutation in human sarcomatoid SDCs and the first transgenic mouse model that recapitulates human SDC sarcomatoid variants. Using this model, we demonstrated the ductal initiation and subsequent development of SDC-like tumors and revealed some of the underlying molecular pathways. Moreover, we propose a link between the KRAS mutation and sarcomatous manifestation of SDCs. In summary, developing and characterizing mouse models that recapitulate human SDC will be a critical step to increase our understanding of these tumors and to discover targets for therapeutic intervention to improve patient outcome. We feel very strongly that our model, with the short latency period and rapid progression, will enable timely research eventually leading to a better understanding of the pathobiology of this relentless and deadly disease.

## Materials and Methods

### Human case selection

This retrospective study was approved by the Institutional Review Boards of Chi Mei Medical Center (IRB 10311005) and was conducted in accordance with the approved guidelines. Salivary gland carcinomas were retrieved from the archive of Department of Pathology at Chi Mei Medical Center and consultation file (CF Li) and reclassified based on the WHO classification, yielding 20 SDCs. To precisely analyze the features of SDC, 2 cases of SDC ex pleomorphic adenoma were excluded. The remaining 18 SDCs with available hematoxylin/eosin (HE), immunohistochemical stains, and paraffin tissue at time of diagnosis were for further analysis of the clinicopathologic and molecular features.

### Screening *EGFR* and *KRAS* mutations

Screening of *EGFR* and *KRAS* mutations was performed by Sequenom MassARRAY platform with OncoFOCUS Panel v1.0 (Supplementary Table S1, Sequenom, San Diego, CA). Mutiplex PCR reaction for tumor DNA (20 ng) was admixed with Taq polymerase (0.2 units), PCR primer (2.5 pmol) and dNTP (25 mM, Sequenom, PCR accessory and Enzyme kit) in 5 μl volume. Thermocycling was programmed as 94  °C for 4-minutes followed by 45 cycles of 94°C for 20-sec, 56 °C for 30-sec and 72 °C for 1-minute, then 72 °C for 3-minutes, respectively. After deactivation of unincorporated dNTPs by using shrimp alkaline phosphatase (0.3 units), single base extension reactions was performed by iPLEX Pro single base extension reactions by using Sequenom, iPLEX Pro kit according to manufacturer’s instructions. After removing residual salt from the reactions by cation-exchange resins, 7 nl of the purified primer extension reaction was loaded onto a matrix pad of a SpectroCHIP (Sequenom, San Diego, CA), analyzed using a MassARRAY Analyzer 4, and the calling by clustering analysis with TYPER 4.0 software.

### Validation of *KRAS* mutations by using pyrosequencing

The analysis of *KRAS* mutation status was performed by using PyroMark Q24 KRAS v2.0 assays to detect *KRAS* codons 12, 13, 61 and 146 mutations. In brief, 2–10 ng of genomic DNA was subjected to PCR reactions. Twenty μl of PCR product was subjected to Pyrosequencing analysis on a Q24 instrument (QIAGEN) by using the CE-IVD marked PyroMark KRAS kit (QIAGEN) according to the manufacturer’s instructions.

### Mouse Breeding

Animal care and experimental procedures were conducted in accordance with guidelines and regulations of the Institutional Animal Care and Use Committees at City of Hope and the University of Texas M. D. Anderson Cancer Center. All mouse colonies were maintained in pathogen-free barrier facilities. *LGL-KRas*^*G12V*^ mice were obtained from the Mouse Models for Human Cancer Consortium Repository (Rockville, MD)^58^, and *Ela-CreERT* transgenic mice^26^ were generated at the Genetically Engineered Mouse Facility of M.D. Anderson Cancer Center and then cross with a pure FVB/129 background strain from Jackson Labs. The Institutional Animal Care and Use Committee at City of Hope and the University of Texas M. D. Anderson Cancer Center approved all animal experiments. *LGL-KRas*^*G12V*^ homozygous and *Ela-CreERT* homozygous mice were crossed at 8-weeks of age to derive *LGL-KRas*^*G12V*^;*Ela-CreERT* mice. *mTmG*^28^ and Rosa26R^29^ homozygous reporter mice were crossed with *Ela-CreERT* homozygous mice at the age of 8-weeks to breed *mTmG;Ela-CreERT* and *Rosa26R;Ela-CreERT* mice, respectively. 8- to 10-weeks old *LGL-KRas*^*G12V*^;*Ela-CreERT, mTmG;Ela-CreERT* or *Rosa26R;Ela-CreERT* mice were gavaged with TAM (3 mg/40 g body weight) or vehicle daily for 5 consecutive days. Tail biopsies were conducted at 3-weeks of age. Tail DNAs were prepared for PCR using DNeasy Kit (Qiagen, Venlo, Netherlands) following manufacture’s protocol. Primer pairs, 5´-ATACCGGAGATCATGCAAGC-3´ (forward) and 5´-ATAGATCATGGGCGGTTCAG-3´ (reverse), were used to generate PCR product for *Ela-CreERT* transgene with a size of 294-base pairs (Bps). Primer pairs, 5´-GCAGCTCGCCGACCACTACCAG (forward) and 5´-TGCCTACGCCAACAGCTCCAACTA-3´ (reverse), were used to screen for *LGL-KRas*^*G12V*^ transgene with a PCR product size of 333-bps. PCR thermocycles as follows: 94 °C for 3-minutes, 35 circles of 94 °C for 1-minute, 60 °C for 1-minute, 72 °C for 1:10-minutes, and 72 °C for 5-minutes.

### Tissue Preparation and Characterization

Salivary parotid, submandibular and sublingular glands were harvested at indicated days post the first day of TAM-feeding. Tumor masses were excised *en bloc* with adjacent tissues. Tissue samples were fixed in 10% neutral-buffered formalin for 48-hours. Tissue embedding, sectioning, and staining with hematoxylin and eosin were performed in City of Hope Pathology Core and MD Anderson Cancer Center Pathology Core. Briefly, after deparaffinization and rehydration, sections were stained with hematoxylin for 4-minutes, rinsed in tap water; and destained with 0.3% acid alcohol. After rinsing in tap water, sections were stained with eosin for 2-minutes, and followed by dehydration, clearing and mounting.

### Primary Salivary Gland Tumor Cells Isolation

Isolation and culture of primary salivary gland tumor cells were as previously described^59^. In brief, day-23 SMG tumors were minced into small pieces and digested in collagenase-hyaluronidase-DNase I buffer for up to 60 minutes at 37 °C on a sterile petri dish. The suspension of digested tumor cells was then passed through a 100-μm sieve (BD) to remove remaining tissue chunks. Red blood cells were lysed by incubating cell suspensions in 1X RBC lysis buffer for 3-minutes on ice. The resulting cells were plated on collagen I coated dishes, and maintained in a salivary gland medium (SGM) consisting of DMEM with FBS (10%), glutamine (5 mM), hydrocortisone (400 ng/ml), insulin (5 μg/ml, Life Technologies), EGF (20 ng/ml, Life Technologies), HEPES (15 mM) and antibiotic-antimycotic (1X, Life Technologies). After 1- to 2-weeks of incubation, colonies of the GFP-negative tumor cells were manually picked and transfer to new cell culture dishes.

### Clonogenic Assay

The SMG tumor cells were seeded on 6-well plates at a density of 1000 cells per well. Cetuximab was added to culture medium at the final concentration of 200 μg/ml and replenished every 2-days. After 5-days of incubation, the colonies were fixed with cold absolute methanol and stained with crystal violet (0.5%). The Images were taken and analyzed with QuantityOne (Bio-Rad).

### Antibodies

Rabbit polyclonal anti-Ki-67 antibody (RB-1510-P) was from Thermo Scientific. Rabbit polyclonal anti-EGFR antibody (ab2430), rabbit anti-smooth muscle actin alpha polyclonal antibody (ab5694), p63 polyclonal antibody (ab53039), p40 polyclonal antibody (ab167612), AR clonal antibody (ab9474), rabbit monoclonal EGFR antibody (ab52894) and phosphoY1068 EGFR antibody (ab40815) were from Abcam. Rabbit anti-elastase I polyclonal antibody was from Life Span BioSciences, Inc. Biotin rat anti-mouse CD45 (53078), rat anti-mouse Ly-6G and Ly-6C (Gr-1) (550291) were from BD Biosciences and anti-mouse F4/80 (14-4801) was from eBioscience. Antibodies against AKT (9272), phosphor-S473 AKT (9271), and phosphor-ERK1/2 (4370) were from Cell Signaling. Anti-ERK1 antibody (sc-93) was purchased from Santa Cruz and anti-ACTIN antibody (MAB 1501R) from Millipore.

### Active RAS pulldown assay

RAS pulldown assay was performed with the RAS-binding domain (RBD) of Raf-1 bound to glutathione agarose beads (Millipore, Cat. #14-278) and the 5X Mg^2+^ Lysis/Wash Buffer (Millipore, Cat. # 20–168), following the manufacturer’s protocol. Briefly, SMG samples were snap-frozen after dissection and grounded in liquid nitrogen, dissolved in the lysis buffer, and then homogenized in tissue homogenizer (Micro-Metric Instrument Company). Equal amount of whole tissue lysates (1 mg) were pre-cleaned with glutathione S-transferase beads, then mixed with 20 μl of Raf1 RBD agarose beads, rotated at 4 °C for 1-hour, washed three times with lysis/wash buffer, boiled for 5-minutes in Laemmli buffer under reducing conditions, and resolved by 12% SDS-PAGE, followed by Western blotting with anti-KRAS antibody (ABD Serotec, Cat. #MCA3223Z).

### Collagen detection

Trichrome staining (Sigma-Aldrich) to reveal collagen deposits in paraffin-embedded submandibular tissue sections was used. Briefly, after deparaffinization, sections were mordanted in pre-heated Bouin’s solution and washed. Slides were then stained in a Weigert’s iron hematoxylin solution, Biebrich scarlet-acid fuchsin, phosphotungstic/phosphomolybdic acid solution, and aniline blue solution and washed in 1% acetic acid. Samples were dried, dehydrated, and coverslip for analysis.

### β-Galactosidase Activity Assay

For detection of β-galactosidase activity in SMGs in mice bearing the *Rosa26 floxed* gene (PMID: 9916792), frozen SMG sections from TAM-fed (or not) *Rosa26R;Ela-CreERT* mice were fixed in formaldehyde (2%)/glutaraldehyde (0.2%), ethylene glycol-bis(β-aminoethyl ether)-N, N, N, N-tetraacetic acid (50 mM) and MgCl_2_ (100 mM) in PBS for 15-minutes. Sections were then washed 3 times with PBS and then stained with an X-gal solution (pH 7.4) containing X-gal (1 mg/mL), potassium ferricyanide (5 mM), potassium ferrocyanide (2 mM), MgCl_2_ (2 mM), sodium deoxycholate (0.01%), and Nonidet P-40 (0.02%) at 37 °C overnight followed by counterstaining with nuclear fast red.

### Immunohistochemistry and Quantization

Immunohistochemistry was performed using Vector Citrate-based Antigen Unmasking Solution, Vectastain Elite ABC Kit, Avidin/Biotin Blocking Kit, and DAB Substrate Kit for Peroxidase (Vector, Burlingame, CA) according to manufacturer’s protocols. Briefly, paraffin sections were deparaffinized and hydrated through xylenes and graded alcohol solutions. Slides were pressure cooked in citrate-based unmasking solution for 30-minutes and washed in PBS for 5-minutes, followed by quenching of endogenous Peroxidase activity in H_2_O_2_ (0.3%) for 30-minutes and blocking for 20-minutes with a mixture of Avidin D solution and diluted normal blocking serum which was prepared from the species in which the secondary antibody is made. Slides were then incubated with a mixture of primary antibody and biotin solution for 30-min and washing in buffer for 3 times. Slides were incubated in the Vector biotinylated secondary antibody for 30-minutes, washed for 5-minutes, and then incubated in Vectastain Elite ABC Reagent for 30-min. After being washed for 5-minutes, slides were processed with DAB Substrate Kit to get desirable staining. Slides were counter-stained with haematoxylin, dehydrated, and mounted. The positive IHC staining in at least five non-overlapping 10X fields was counted with Image-Pro Premier 9.0 (Media Cybernetics). Immunohistochemical staining for leukocytes (CD45 and Gr-1) and macrophages (F4/80) was performed on frozen salivary gland sections. After fixing in pure acetone at −20 °C for 10-minutes followed by blocking with normal horse serum (10%) for 60-minutes at room temperature, primary antibodies were applied and incubated at 4 °C overnight. Sections were washed and incubated with biotinylated secondary antibodies except for CD45 and streptavidin-labeled horseradish peroxidase. Positive labeling was detected using the NovaRED substrate kit for peroxidase. Counterstaining was performed with Gill no. 3 hematoxylin.

### GFP and RFP Visualization

Mouse SMG samples from TAM-fed (or not) *mTmG;Ela-CreERT* mice were fixed with paraformaldehyde (4%) in PBS solution in 4 °C overnight. Samples were rinsed with 1X PBS for half-hour at room temperature, followed by dehydration in 15% sucrose and 30% sucrose, respectively in room temperature till samples sink down. After being immersed in sucrose (30%) and Optimal Cutting Temperature compound (O.C.T., 50%, Tissue-Tek) in 4 °C overnight, samples were embedded in O.C.T. on dry ice. Section of 10 μm were air-dried, fixed with 4% paraformaldehyde for 10-minutes at room temperature and then washed with PBS for 5-minutes (3X) to remove O.C.T. Sections were mounted on slides with mounting media and then viewed under Olympus IX81 automated inverted fluorescence microscopy. Fluorescence images of sample sections vs. controlled tissue section were taken with identical exposure time. At least three images were taken for each section and fluorescence, and representative images are shown.

### RNA Extraction, qRT-PCR, and RNA-seq

Total RNAs was prepared from excised tissue samples with Trizol Reagent (Life technologies) following manufacturer’s instructions. The concentration and purity of RNA were measured by NanoDrop spectrophotometry (NanoDrop Technologies Inc., LLC). RNA-seq was performed by City of Hope DNA Sequencing Core. Gene Set Enrichment Analysis was performed using the software of the Broad Institute. Gene ranking was performed based on differences between naive SMGs and SMG tumors. Sets were considered enriched if the false discovery rate (FDR) was less than 25% with a nominal *p* < 0.01.

### Cetuximab Treatment

Clinical grade cetuximab at a concentration of 2 mg/ml was supplied by Bristol-Myers Squibb. At the sixth day of TAM-gavage, randomly grouped *LGL-KRas*^*G12V*^;*Ela-CreERT* mice received cetuximab (1 mg/mouse, *i.p.*, twice a week) or vehicle until mice were euthanized.

### Statistical Analysis

All results are given as mean ± SD of at least three independent experiments. Student *t* tests and one-way analysis of variance (ANOVA) were used to ascertain statistical significance. Statistical significance was set as *p* < 0.05, two-tailed.

## Additional Information

**How to cite this article**: Fu, Y. *et al*. Ductal activation of oncogenic KRAS alone induces sarcomatoid phenotype. *Sci. Rep*. **5**, 13347; doi: 10.1038/srep13347 (2015).

## Supplementary Material

Supplementary Information

## Figures and Tables

**Table 1:** Clinicopathological and molecular features of 18 salivary ductal carcinoma enrolled.

Case No.	Age	Gender	Location	Operation	Stage	Histological variant	Mutation
SDC-01	59	M	Right Submandibular Gland	Wide Excision	T3N2bM0	—	—
SDC-02	58	M	Left Parotid Gland	Total Parotidectomy	T3N2bM0	—	—
SDC-03	59	F	Right Parotid Gland	Total Parotidectomy	T4aN0M1	—	—
SDC-04	57	M	Left Parotid Gland	Total Parotidectomy	T3N0M0	—	—
**SDC-05**	**41**	**F**	**Left Parotid Gland**	**Total Parotidectomy**	**T3N2bM0**	**Sarcomatoid (10%–15%)**	***KRAS*** **p.A146T** (AGC>AAC), Frequency: 12%
SDC-06	63	M	Left Submandibular Gland	Submandibulectomy	T3N2aM0	—	—
SDC-07	53	F	Left Parotid Gland	Superficial lobectomy	T1NxM0	Micropapillary (60%)	—
SDC-08	59	M	Right Submandibular Gland	Submandibulectomy	T3NxM1	—	—
SDC-09	85	M	Left Parotid Gland	Total Parotidectomy	T2N0M0	—	—
SDC-10	49	M	Right Parotid Gland	Tumor Excision	T1N0M0	—	—
**SDC-11**	**62**	**M**	**Left Parotid Gland**	**Total Parotidectomy**	**T4aN2bM0**	**Sarcomatoid (10%)**	***KRAS*** **p.Q61H** (CAA>CAC), Frequency: 9.5%
SDC-12	57	F	Left Parotid Gland	Tumor Excision	T2N1M0	Sarcomatoid (30%)	—
SDC-13	48	M	Left Parotid Gland	Wide Excision	T3N0M0	Mucinous (75%)	—
SDC-14	48	M	Right Submandibular Gland	Wide Excision	T3N2bM1	—	—
SDC-15	71	F	Right Submandibular Gland	Wide Excision	T2N1M1	—	—
SDC-16	72	F	Right Parotid Gland	Total Parotidectomy	T3N2bM0	Sarcomatoid (25%)/micropapillary (10%)	—
SDC-17	42	M	Right Parotid Gland	Superficial Parotidectomy	T1N0M0	—	—
SDC-18	81	M	Left Parotid Gland	Total Parotidectomy	T4N0M0	—	—
