# Supplementary material for: Ductal activation of oncogenic KRAS alone induces sarcomatoid phenotype
Source: Sci Rep. 2015 Aug 20;5:13347. doi: 10.1038/srep13347 (PMC4642517; doi:10.1038/srep13347)
Supplement: Supplementary Information [file srep13347-s1.pdf]

Ductal activation of oncogenic KRAS alone induces sarcomatoid phenotype

Yong Fu, Zobeida Cruz-Monserrate, H. Helen Lin, Yiyin Chung, Baoan Ji, Szu-min Lin, Steven Vonderfecht, Craig D. Logsdon, Chien-Feng Li, David K. Ann

Supplementary Data

Supplementary Figure Legends

Supplementary Fig. S1. Pyrosequencing validation confirms the presence of somatic KRAS A146T mutation featured by GCA>ACA in 12% tumor DNA submitted (***A***) and Q61H mutation showing CAA>CAC mutation in 9.5% (***B***), respectively.

Supplementary Fig. S2. The experimental design for TAM gavage feeding and SMG harvesting. TAM was gavaged for five consecutive days. Salivary gland and tumor were sampled at day-6, -9, -12, -15, and -24.

Supplementary Fig. S3. Significant increase in leucocytes and macrophages infiltration in SMG tumors. (*A*) Immunohistochemical staining for CD-45, Gr-1 and F4/80 is shown of SMGs in TAM-fed *LGL-KRas^{G12V};Ela-CreERT* mice on day-15 following tumor induction. **(*B*)** Mouse SMG sarcomatoid tumor cells were p63 (*top panel*)- and p40 (*middle panel*)-negative. The residual myoepithelium in SMGs served as the respective positive internal control (arrows). Consistent with Fig. 1, these oncogenic KARS-expressing sarcomatoid tumor cells are negative for AR expression (*bottom panel*). Scale bar: 200 μm.

Supplementary Fig. S4. The histopathology of KRAS^{G12V}-induced tumors. (*A*) Multiple morphological manifestations during day-9 (*panel a, b*) and day-12 (*panels c-f*) suggested the ductal origin of the KRAS^{G12V}-induced SMG tumors. Scale bars 20 μm (*panels a - e*); 50 μm (*panel f*). G: GCT; A: acini; S: sarcomatous element; SD: striated duct; ED: excretory duct. **(*B*)** Masson's trichrome staining for collagen deposition analysis of SMGs from *LGL-KRas^{G12V};Ela-CreERT* mice at 15-days post TAM-feeding.

Supplementary Fig. S5. Early morphological manifestation reflecting tumorigenesis of SMGs. Initial dysplasia (black arrow) was only observed in ductal cells near or at striated duct

(green arrow) in close proximity to blood vessel (red arrowhead) on day-9 post oncogenic KRAS induction. Five randomly selected images are shown. Scale bar: 100 μm (*panels a - e*); 200 μm (*panel f*).

Supplementary Fig. S6. Abnormal parotid and sublingual salivary glands of Tam-fed *LGL-KRas^{G12V};Ela-CreERT* mice. (***A***) Parotid tumors on day-18 post TAM-feeding. Advanced sarcomatoid carcinoma with necrosis (*panel a*, black arrow; scale bar: 200 μm), without recognizable structure (*panel b;* scale bar: 100 μm) and with typical sarcomatous changes (*panel c;* scale bar: 50 μm): high nucleus-to-cytoplasm ratio (*panel c*), giant and bizarre nucleus (*panel c*, black arrow), multiple nuclei (*panel c*, red arrow), and spindle cells (*panel c*, red arrowhead). (***B***) Sublingual tumors on day-15 post TAM-feeding. Dysplasia at striated ducts (*panel a*, black arrow; scale bar: 50 μm), microinvasive carcinoma (*panel b*, black arrow; scale bar: 50 μm), dysplasia at a striated duct (*panel c,* black arrow; scale bar: 20 μm.

Supplementary Fig. S7. Close proximity of nuclear Ki-67-positive staining cell with blood vessel. A dyad location of striated duct (green arrow) and a blood vessel (red arrowhead) to the nuclear Ki-67-positive staining cell in the striated duct (black arrow) on day-9. There was no Ki-67-positive staining in the adjacent region to blood vessel alone (red arrow). Scale bar: 100 μm.

Supplementary Fig. S8. Gland/tumor weight measured on day-24 post TAM-feeding. (***A***) Tumor weight is significantly different between female and male mice on day-24 post TAM-feeding. 5 mice of the same age were included in each group. (***B***) Statistic analysis shows a significant increase of gland weight comparing to vehicle-fed control. However, cetuximab treatment did not significantly reduce tumor size. Data is shown as mean ± SD, n ≥ 3. Data is analyzed with Graphpad Prism 6 using non-parametric Mann-Whitney test. *: $p < 0.05$, **: $p < 0.01$. (***C***) Cetuximab fails to inhibit SMG tumor cell colony formation *in vitro*. Tumor cells isolated from day-23 SMG tumors were seeded on 6-well plates at a density of 1000 cells per well and treated with cetuximab (200 μg/ml) for 5-days. One set of representative images is shown. Quantification of colony numbers is shown as mean ± SD of three independent experiments. (***D***) Cetuximab inhibits EGF-induced Akt and ERK signaling *in vitro*. SMG tumor cells were maintained under a serum-starved condition (DMEM with 2% FBS) in the presence or absence of cetuximab (200 μg/ml) for 72-hours. Cells were then challenged with EGF (100 ng/ml) for 5-minutes prior to cell lysis and Western blot analyses. One representative Western blot is shown. (***E***) Cetuximab does not inhibit Akt and ERK signaling in SMG tumor cells *in vitro*. Isolated tumor

cells cultured under normal growth conditions were treated with cetuximab (200 μg/ml) for 48- or 72-hours and subjected to Western blot analyses as indicated. One representative Western blot is shown.

Supplementary Table S1. Detectable *EGFR* and *KRAS* mutations in Sequenom OncoFOCUS Panel

Gene Name	No. of mutation type detected	Types of mutations
EGFR	128	A289D, A289V, A767_S768insTLA, D761N, D761Y, D770_N771>AGG, D770_N771insAPW, D770_N771insG, D770_N771insGD, D770_N771insGF, D770_N771insGL, D770_N771insMATP (5'Detection Only), D770_N771insSVD, D770_P772>ASVDNR, D770>GY, D770N,E709A, E709fs*1, E709G, E709H, E709K, E709Q, E709V, E746_A750>DP, E746_A750>IP, E746_A750>QP, E746_A750>RP, E746_A750>VP, E746_A750delELREA, E746_E749del, E746_E749delELRE, E746_P753>IS, E746_P753>LS, E746_P753>VQ, E746_P753>VS, E746_S752>A, E746_S752>D, E746_S752>I, E746_S752>V,E746_S752delELREATS, E746_T751>A, E746_T751>I, E746_T751>IP, E746_T751>L, E746_T751>Q, E746_T751>S, E746_T751>V, E746_T751>VA, E746_T751>VP, E746_T751delELREAT, E746K, E746V, G598V, G719A, G719C, G719D, G719S, H773_V774insH, H773_V774insNPH, H773_V774insPH, H773_V774insQ, H773>NPY(3' Detection Only), I759N, K745_E749delKELRE, L747_A750>P, L747_A755>AN, L747_ E749del, L747_ E749delLRE, L747_ K754>ST, L747_ K754delLREATSPK, L747_ P753>Q, L747_ P753>S, L747_ S752>Q, L747_S752>QH, L747_ S752delLREATS, L747_T751>A , L747_T751>P, L747_T751>PT, L747_T751>Q, L747_T751>S, L747_T751del, L747_T751delLREAT, L747P, L747S, L858K, L858M, L858R, L861Q, L861R, M766_A767insAI, N771_P772>SVDNR, N771_P772insRH, N771>GF, N771>GY,N771>SH, N771>TH, P753_I759del, P753_I759delPKANKEI, P753Q, P753S, R108K, R776C, R776H, S752_I759delSPKANKEI, S752P, S752Y, S768_V769>IL, S768I, S768N, S768T, T263P, T751_E758delTSPKANKE, T751_I759>N, T751_I759>REA, T751_I759>S, T751fs*4, T751I, T790M, T854A,V769_D770insASV, V769_D770insCV, V769_D770insGSV, V769_D770insGVV, V774_C775insHV, V774L, V774M
KRAS	37	A146G, A146P, **A146T**, A146V, A59T, G12A, G12C, G12D, G12E, G12F, G12G, G12I, G12L, G12N, G12P, G12R, G12S,G12T, G12V, G12W, G12Y, G12_G13insA, G13_V14insG, G13A, G13C, G13D, G13I, G13N, G13R, G13S, G13V, Q61E, **Q61H**, Q61K, Q61L, Q61P, Q61R
NRAS	20	G12A, G12C, G12E, G12N, G12P, G12V, G12Y, G13A, G13D, G13N, G13R, G13V, G13Y, Q61E, Q61H, Q61K, Q61L, Q61P, Q61Q, Q61R
BRAF	19	D594G, D594V, G469A, G469E, G469R, G469S, G469V, L597Q, L597R, L597S, L597V, T599_V600insT, T599_V600insTT,V600E, V600K, V600L, V600M, V600R, V600>YM

A

SDC-05 *KRAS* p.A146T

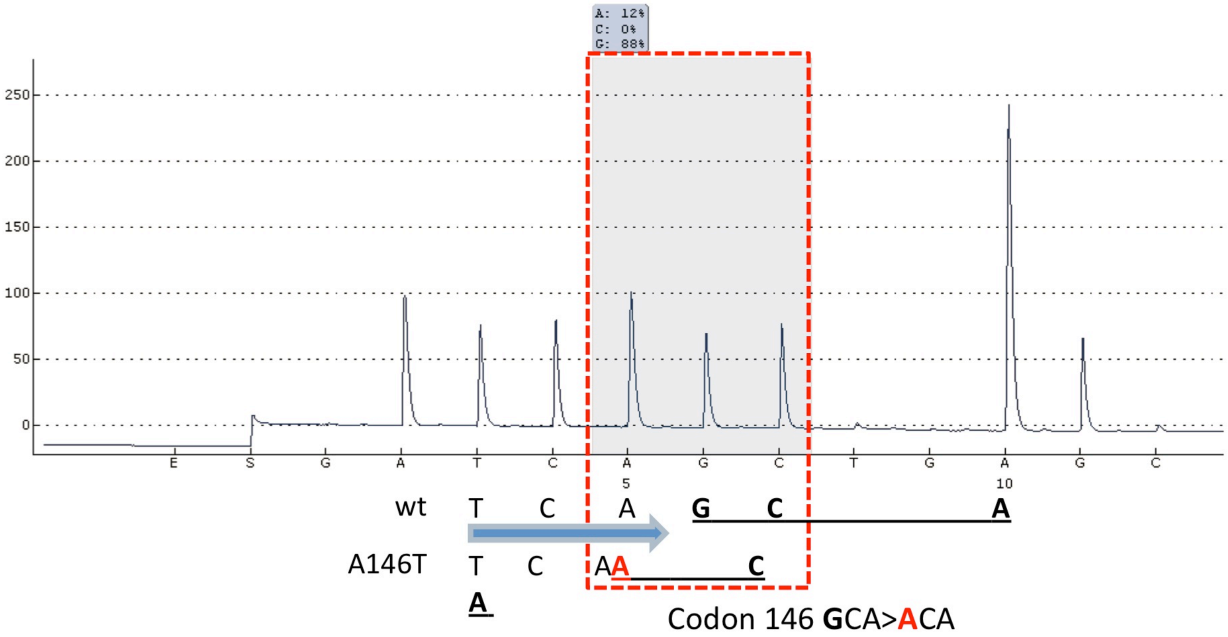

B

SDC-11 *KRAS* p.Q61H

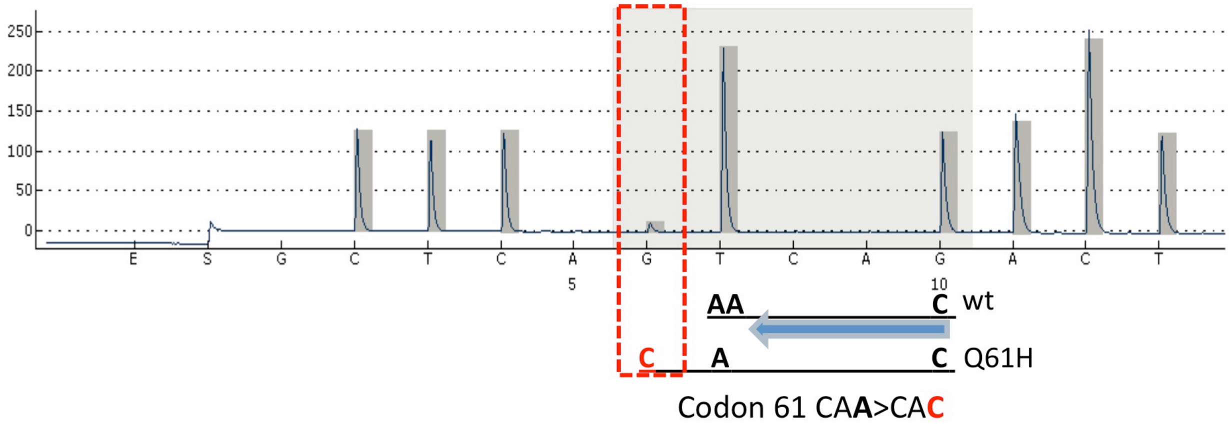

Supplementary Figure S1

Experimental Protocol

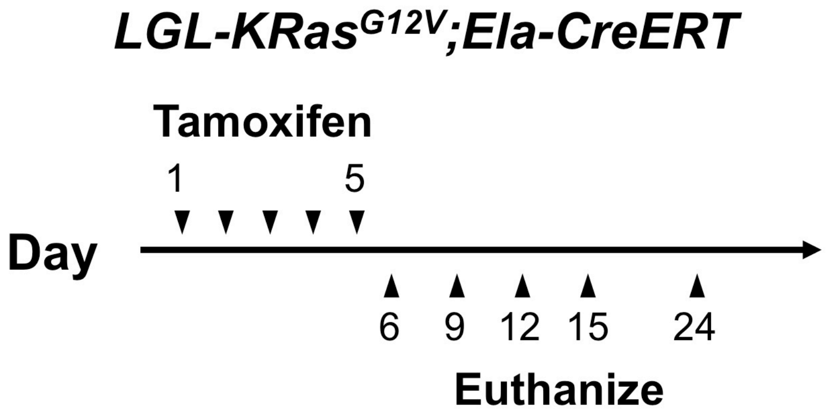

Supplementary
Figure S2

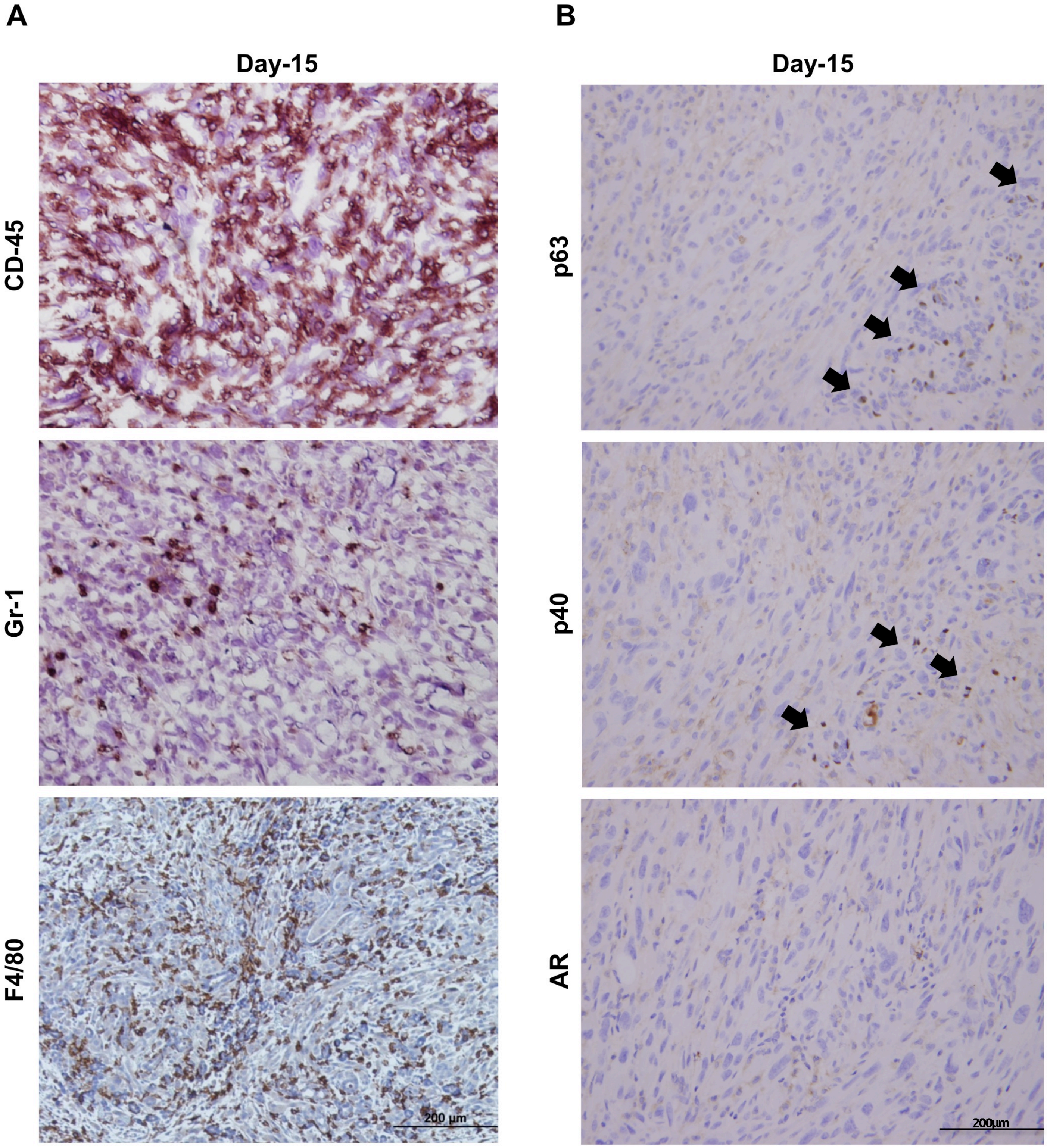

Supplementary
Figure S3

A

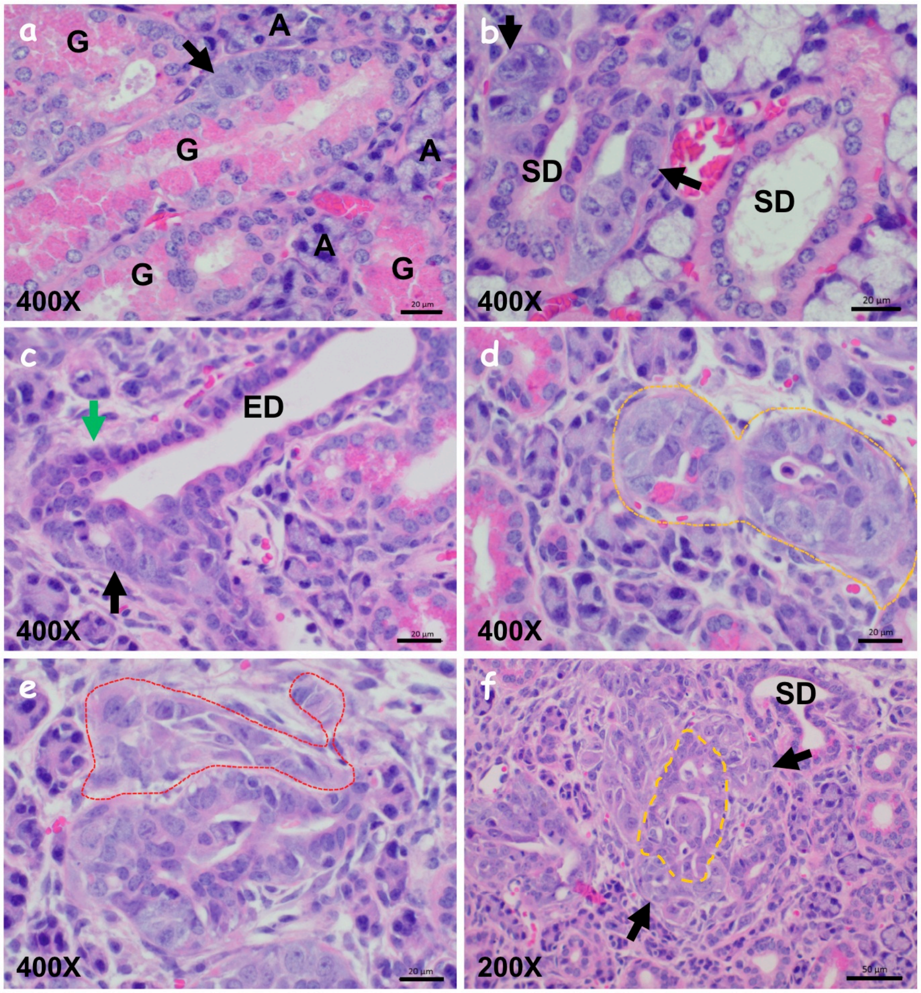

B

Trichrome Staining

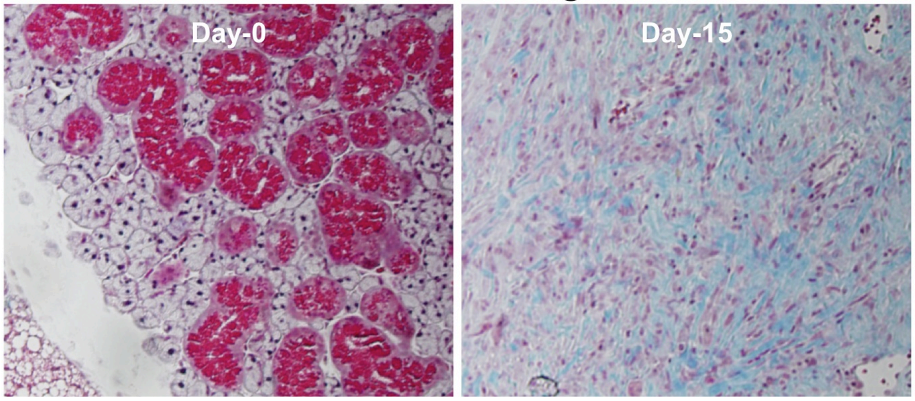

Supplementary Figure S4

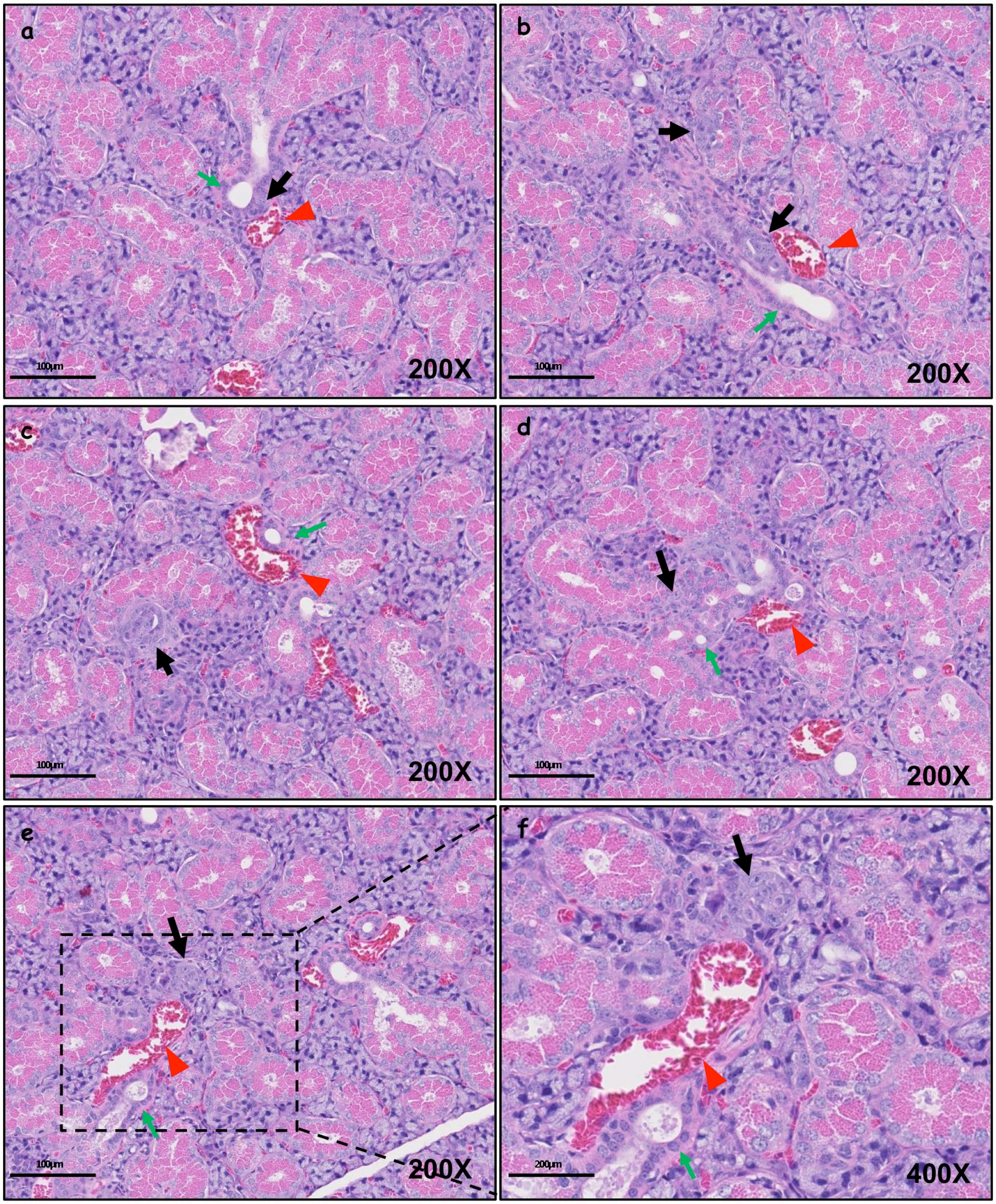

Supplementary Figure S5

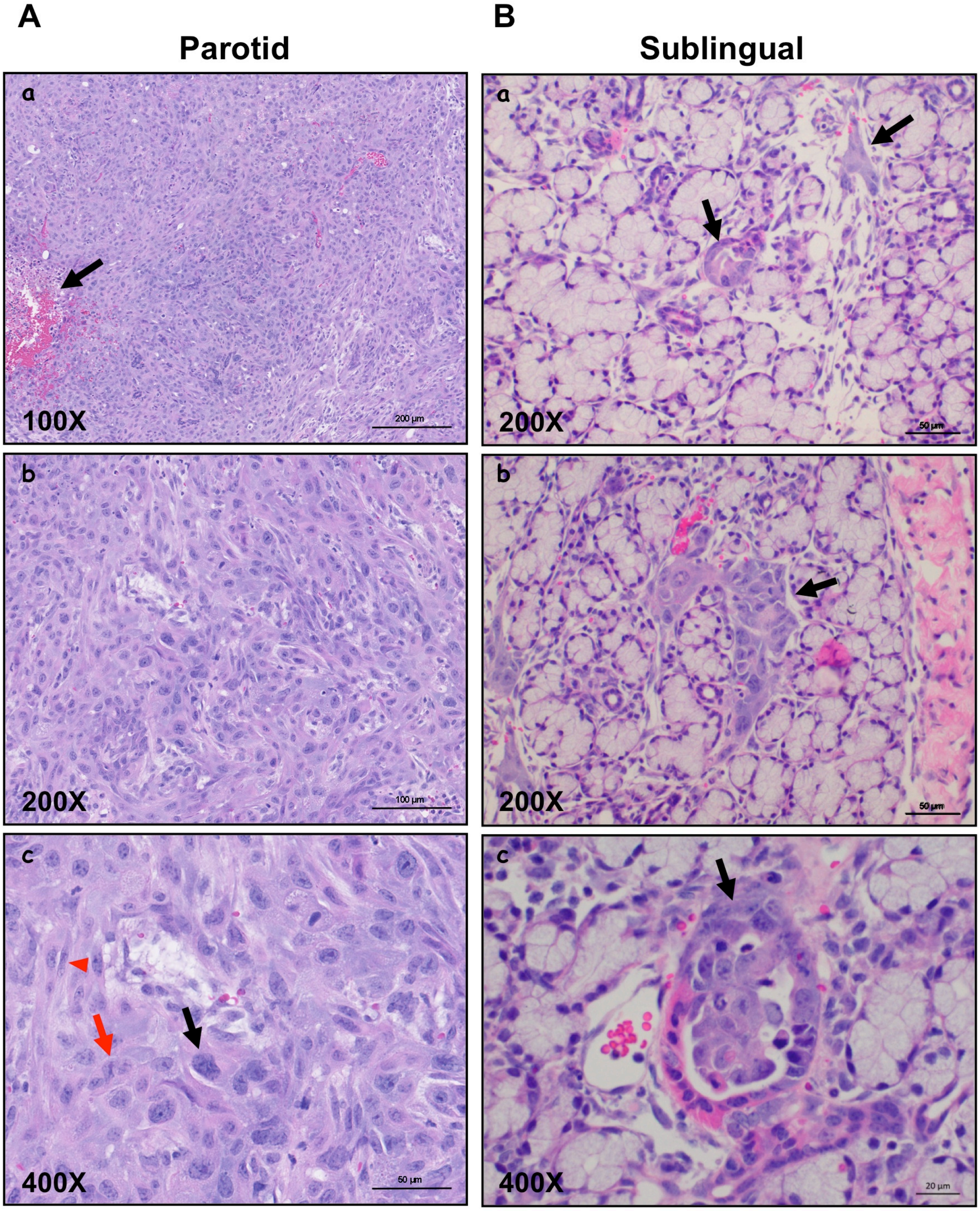

Supplementary Figure S6

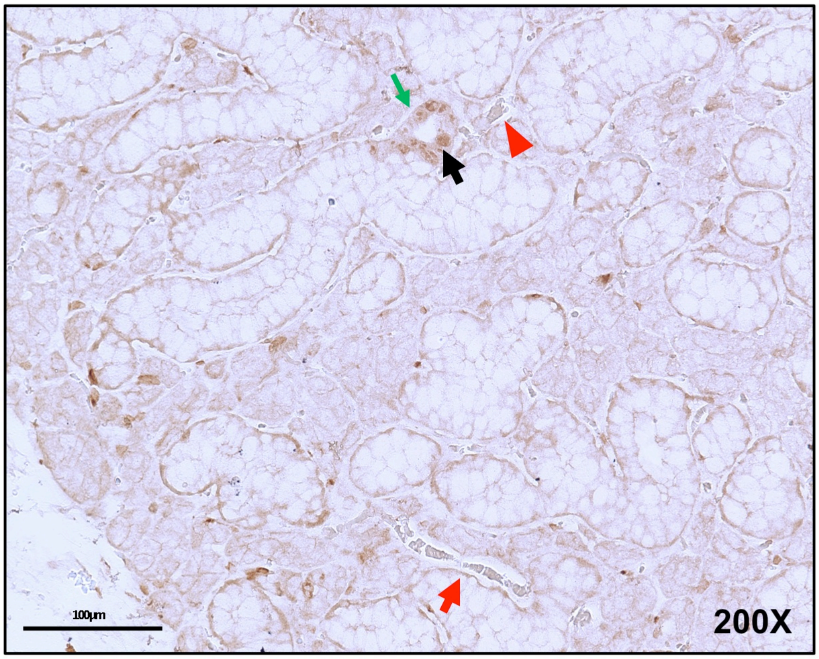

Supplementary Figure S7

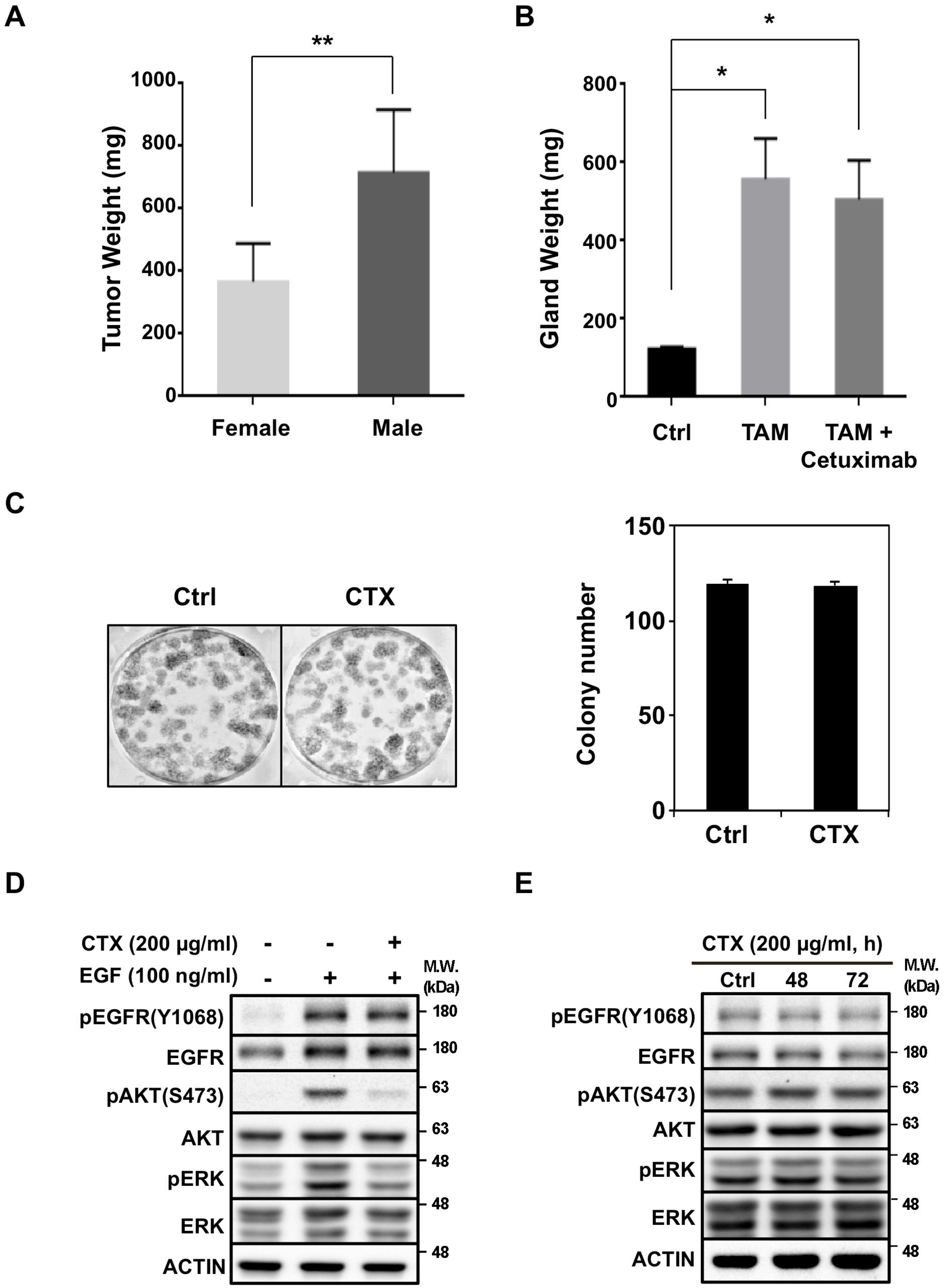

Supplementary Figure S8
